# Characterization of *Rosa canina* Fruits Collected in Urban Areas of Slovakia. Genome Size, iPBS Profiles and Antioxidant and Antimicrobial Activities

**DOI:** 10.3390/molecules25081888

**Published:** 2020-04-19

**Authors:** Katarína Rovná, Eva Ivanišová, Jana Žiarovská, Peter Ferus, Margarita Terentjeva, Przemysław Łukasz Kowalczewski, Miroslava Kačániová

**Affiliations:** 1 Department of Planting Design and Maintenance, Faculty of Horticulture and Landscape Engineering, Slovak University of Agriculture, Tr. A. Hlinku 2, 94976 Nitra, Slovakia; katarina.rovna@uniag.sk; 2 Department of Technology and Quality of Plant Products, Faculty of Biotechnology and Food Sciences, Slovak University of Agriculture, Tr. A. Hlinku 2, SK-94976 Nitra, Slovakia; eva.ivanisova@uniag.sk; 3 Department of Plant Genetics and Breeding, Faculty of Agrobiology and Food Resources, Slovak University of Agriculture, Tr. A. Hlinku 2, 94976 Nitra, Slovakia; jana.ziarovska@uniag.sk; 4 Department of Dendrobiology, Institute of Forest Ecology, Slovak Academy of Sciences, Ľ. Štúra 2, 96053 Zvolen, Slovakia; peter.ferus@savba.sk; 5 Institute of Food and Environmental Hygiene, Faculty of Veterinary Medicine, Latvia University of Life Sciences and Technologies, K. Helmaņaiela 8, LV-3004, Jelgava, Latvia; margarita.terentjeva@llu.lv; 6 Institute of Food Technology of Plant Origin, Poznań University of Life Sciences, 31 Wojska Polskiego St., 60-624 Poznań, Poland; przemyslaw.kowalczewski@up.poznan.pl; 7 Department of Fruit Sciences, Viticulture and Enology, Faculty of Horticulture and Landscape Engineering, Slovak University of Agriculture, Tr. A. Hlinku 2, 94976 Nitra, Slovakia; 8 Department of Bioenergy, Food Technology and Microbiology, Institute of Agriculture Sciences, Land Management and Environmental Protection, University of Rzeszow, 4 Zelwerowicza St., 35601 Rzeszow, Poland

**Keywords:** *Rosa canina*, bacteria, microscopic fungi, urban area, carotenoids, polyphenols

## Abstract

The studies of plant bacterial endophytes, colonizing the plant tissues without any signs of diseases, are essential for understanding of ecological interactions. The aim of our study is to detect microbiological contamination and to assess the antimicrobial, antioxidant activity, total phenolic, carotenoid content, genome size, and ploidy of non-cultivated *Rosa canina* sampled from urban areas. Samples of *Rosa canina* fruits were collected in three locations in Slovakia. The highest total viable count and the *Enterobacteriaceae* count in fruits were 4.32 log CFU/g and 4.29 log CFU/g, respectively. Counts of the mesophilic anaerobic sporulating bacteria, *Pseudomonas* spp., and of the microscopic fungi and yeasts were 3.00, 2.15 log CFU/g, 3.65 log CFU/g, and 2.76 log CFU/g, respectively. Regarding the antimicrobial activity, *Escherichia coli* and *Klebsiela oxytoca* were the most sensitive species among the assayed microorganisms to the treatment with the ethanolic extracts of *Rosa canina* fruits. The fruits were rich in bioactive compounds, polyphenols, and carotenoids, that could be related to their antioxidant activity. Genome sizes of analyzed samples ranged from 2.3 to 2.96. DNA-based fingerprinting obtained by iPBS markers of the *Rosa canina* var. *lapidicola* Heinr. Braun., was characterized by some distinctive inserted loci. An interdisciplinary study was performed for the dog roses from different parts of Slovakia that resulted in deeper characterization of this species.

## 1. Introduction

Endophytic bacteria colonize the plant tissue without apparent signs of diseases in the host plants. They are ubiquitous in plants and were isolated from roots, leaves, stems, flowers, fruits, and seeds [1]. Endophytic bacteria may influence the plant metabolism, promoting the growth, facilitating the control of soil-borne pathogens, or responding to the host plant to environmental stresses [2,3,4]. Plants and bacteria interactions enhance the plants settlement during ecosystem restoration [5]. 

The internal tissue of healthy plants has been reported host more than 129 bacterial species of 54 genera. *Pseudomonas, Bacillus, Enterobacter,* and *Agrobacterium* were the most common. Earlier reports defined the endophytic bacteria as contamination stemming from an incomplete surface disinfestation or latent pathogens infection, whereas recent studies proved that endophytes enhance plant growth and reduce an impact of several plant pathogens by reducing their disease signs [6].

Application of bioactive compounds of natural origin has a long tradition. They have been widely used in traditional medicine worldwide as well as in treatment and prevention of certain disorders [7,8,9,10,11]. Ongoing research indicates that the flowers of *Rose white* exhibit antioxidant and antimicrobial activity [12], and that the extracts from their petals show activity against bacteria pathogenic for humans [13]. Moreover, rosehips fruits are a rich source of various phenolic compounds (phenolic acids, tannins, anthocyanidins, and flavonoids), vitamins, tocopherols, carotenoids, ascorbic acid, citric acid, malic acid etc., fatty acids, pectins, and sugars. Available data indicate that the high content of polyphenols and vitamins in rose fruit share antioxidant, anticancerogenic and anti-inflammatory properties [14,15]. 

*Rosa canina* L. (dog rose) is a shrub species widespread in Europe that has been grown massively in the past in open landscape as well as in gardens. It is cultivated mainly for the ornamental purposes and remains popular because of its high tolerance to drought. The plant is commonly encountered in the urban areas. The *Caninae* DC. consists of large and well-defined group of polyploid taxa where pentaploids are most common [16,17]. The biological properties of dog rose, including antioxidant and antimicrobial activity, are not well established. Therefore, the aim of the present study are (i) to assess the antimicrobial and antioxidant properties of *Rosa canina* fruits, (ii) to analyze the contents of the endophytic microbiome population that colonize the fruits, and (iii) to determine the genome size and ploidy level in the samples obtained from urban areas in Slovakia.

## 2. Results and Discussion

**Figure 1:**
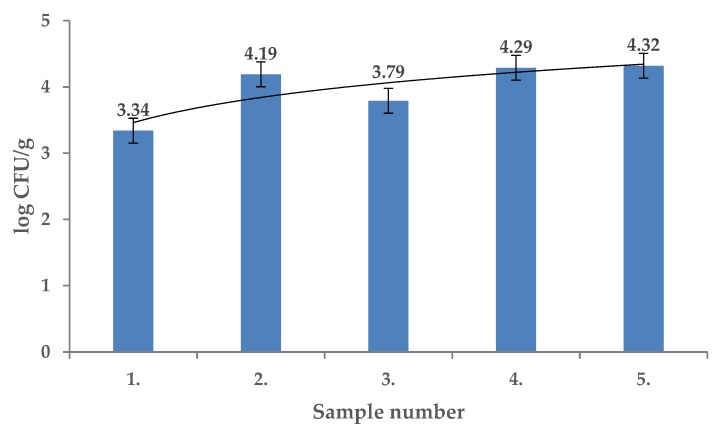
Total viable counts (TVC) in fruits of *Rosa canina.*

**Figure 2:**
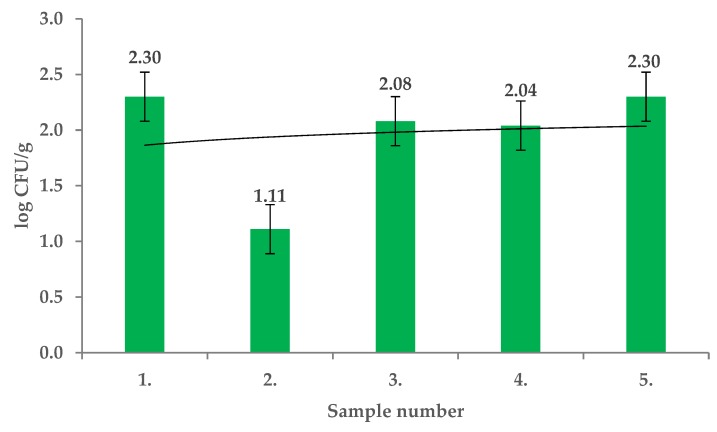
Mesophilic anaerobic sporulating bacteria (MASB) in fruits of *Rosa canina*.

Total viable counts of the endophytic microflora (TVC) of the tested fruits of *Rosa canina* were from 3.34 ± 0.01 log CFU/g to 4.32 ± 0.01 log CFU/g and the differences in contamination were significant among the locations sampled (*p* < 0.01) (Figure 1). The *R. canina* microflora were identified using MALDI TOF MS Biotyper and included *Staphylococcus chromogenes, Sphingobacterium mizutaii, Comamonas aquatica, Bacteroides uniformis, Arthrobacter arilaitensis, Kocuria rosea, Pseudomonas rhodesiae, Arthrobacter arilaitensis, Staphylococcus warneri, Pseudomonas graminis, Streptomyces griseus, Lactobacillus paracasei*, and *Lactobacillus perolens*. The mesophilic anaerobic sporulating bacteria (MASB) counts in fruits of *R. canina* ranged from 1.09 ± 0.08 log CFU/g to 3.00 ± 0.04 log CFU/g and the differences were significant (*p* < 0.01) (Figure 2). From this group *Clostridium hathewayi, Clostridium difficile, Clostridium cochlearium* and *Aromatoleum terpenicum* were isolated and identified. 

The prerequisites for endophytes studies in the plant tissues are sterility of the plant surface and correct choice and application of the isolation methods [6,14]. The culturable endophytic bacteria counts of the *Rosa* plants notoriously vary between the isolation sites [5]. The flowers were comparably less contaminated (1.2 × 10^2^ CFU/g) than the leaves (2.4 × 10^3^ CFU/g), but the roots and stems were colonized heavily (4.6 × 10^4^ and 3.1 × 10^4^ CFU/g, respectively). The contamination pattern shows that the lower plant organs host a higher number of endophytes [18]. Notably, colonization rates with endophytes was different for the same locality in the study of Rovna et al. [19].

Large diversity of plants, which were colonized by endophytic microorganisms, and isolated microorganisms were found [20,21]. Previous results on the bacteriological contamination and diversity of the endophytic microorganisms were in accordance with our study. The present study shows the characteristics of culturable endophytic bacteria of *Rosa canina* grown in Slovakia and may be helpful to select the microorganisms for improving the growth of plants in the present habitat.

**Figure 3:**
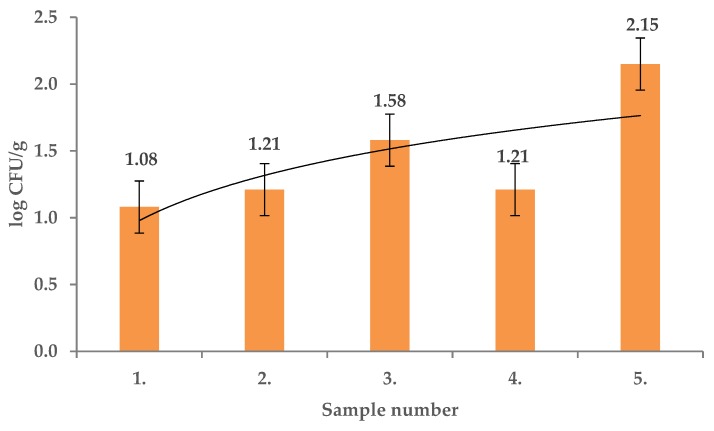
*Pseudomonas* spp. in fruits of *Rosa canina.*

*Pseudomonas* spp. counts in *R. canina* fruits recorded in this study ranged from 1.08 ± 0.01 log CFU/g to 2.15 ± 0.01 log CFU/g (Figure 3) and significant differences between the sample contamination were found (*p* < 0.01). *Pseudomonas rhodesiae* and *Pseudomonas graminis* were identified. *Bacillus* and *Pseudomonas* were previously frequently reported as endophytes, detected using simple cultivation methods [22]. *Pseudomonas* species could be differentiated with routine microbiological methods but MALDI TOF identification is a more accurate tool in identification of certain microorganisms and gives a comprehensive view on the *Pseudomonas* species which may colonize *R. canina* [23].

**Figure 4:**
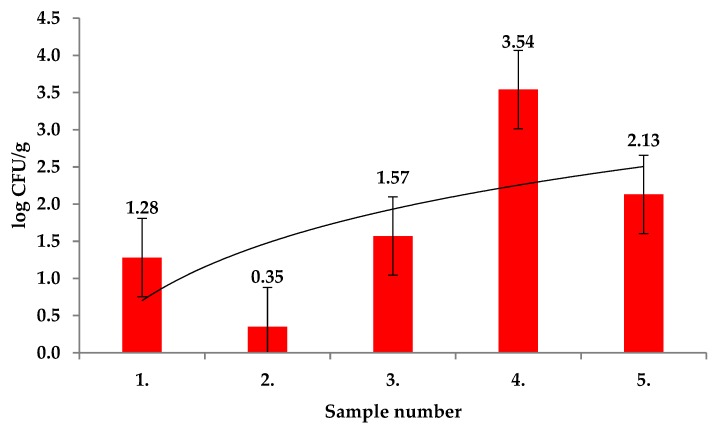
Enterobacteriaceae in fruits of *Rosa. canina*.

Enterobacteriaceae counts in *Rosa canina* fruits ranged from 0.42 ± 0.12 log CFU/g to 3.54 ± 0.01 log CFU/g and differences were significant with *p* < 0.01 (Figure 4). Among the Enterobacteriaceae, *Pantoea agglomerans* and *Erwinia amylovora* were identified using MALDI TOF MS Biotyper in all tested samples. In previous studies, partial 16S rDNA sequences of Enterobacteriaceae allowed to identify *Pantoea agglomerans*, *Klebsiella terrigena*, *Erwinia rhapontici, Rahnella aquatilis* in the leaves of *R. rugosa*. These phyllosphere bacteria showed their distinct metabolic abilities toward plant phenolic compounds [24].

**Figure 5:**
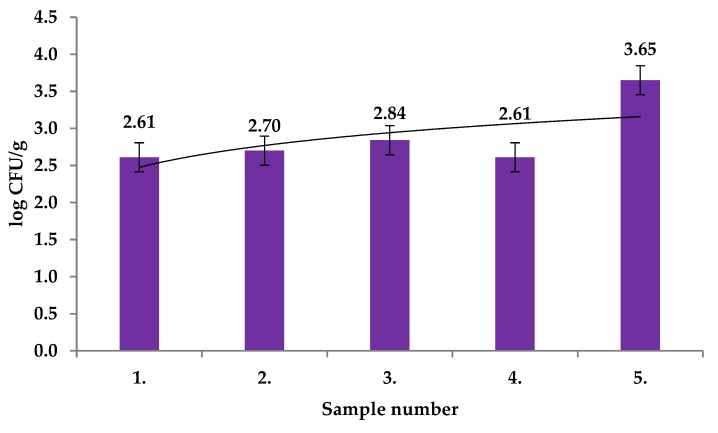
Yeasts counts in fruits of *Rosa canina.*

Yeasts counts in the fruits of *R. canina* collected in this study ranged from 2.61 ± 0.03 log CFU/g to 3.65 ± 0.07 log CFU/g (Figure 5) with significant differences between the samples (*p* < 0.01). *Candida lusitaniae, Candida parapsilopsis,* and *Rhodotorula mucilaginosa* were identified with MALDI TOF MS Biotyper in all tested samples. Yeasts were isolated from the internal tissues of succulent fruits. During ripening, comparable yeast species were found in internal and external tissues of fruit. Moreover, the yeast counts were significantly higher after fruit ripening [25].

**Figure 6:**
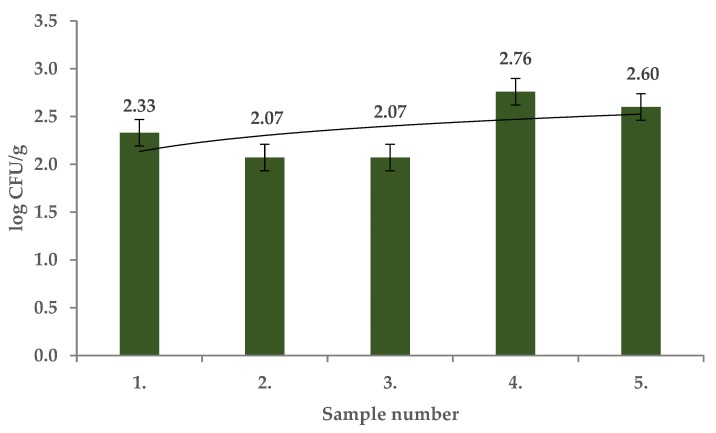
Microscopic fungi in fruits of *Rosa canina*.

Microscopic fungi counts in the present study ranged from 2.07 ± 0.06 log CFU/g to 2.59 ± 0.13 log CFU/g (Figure 6) and significant differences between the samples were identified (*p* < 0.01). The presence of *Mucor circinelloides, Mucor ramosissimus, Epicoccum nigrum, Phoma glomerata, Phoma sorghina, Trichoderma koningii, Penicillium citrinum, Alternaria alternata, Aspergillus versicolor,* and *Cladosporium* spp. was confirmed using MALDI TOF MS. 

Antimicrobial activity of the ethanolic extracts of *R. canina* fruits was evidenced against *Escherichia coli* but not against *Clostridium perfringens* (Table 1). Previous studies document that methanolic extracts of fruits of various *Rosa* spp. plants showed varying antibacterial activities against microorganisms. 

Extract of R. pisiformis extract altered the growth of Yersinia enterocolitica, Streptococcus aureus, Bacillus cereus, and Salmonella typhimurium but R. canina and R. villosa of Enterococcus faecalis and Bacillus cereus. Rosa canina and R. dumalis subsp. antalyensis extracts inhibited growth of Yersinia enterocolitica, but R. dumalis subsp. antalyensis also against that of Bacillus cereus and Klebsiella pneumoniae [26].

The results of the minimal inhibition concertation (MICs) of *R. canina* fruits extracts show that the lowest MIC of the extract was against *E. coli* (32 µg/mL) followed by *K. pneumonia* of 64 µg/mL (Table 2). The extracts were effectively inactive against *C. perfringens* growth. Previously, the antimicrobial activity of *R. canina* leaf extract was evidenced against *P. aeruginosa* and *S. typhimurium* [27]. In another study with *R. canina*-mediated biogenic silver nanoparticles, MIC and antibacterial activity ranged between 16 μg/mL and 256 μg/mL for *Bacillus cereus, Enterococcus hirae, Staphylococcus aureus, Escherichia coli, Legionella pneumophila, Candida albicans*, and *P. aeruginosa* [28]. MIC of methanolic extract of *R. canina* was between 256 and 512 mg/mL against MRSA and MDR bacterial strains [29]. Methanolic extracts of *R. canina* exhibited the strongest antimicrobial activity against *S. aureus* and *C. albicans* with MIC of 2.0 mg/mL. Antimicrobial activity was demonstrated for the water extracts against Gram-positive and Gram-negative bacteria with the lowest MIC of 3.5 mg/mL for *S. aureus*. Acetone extract was effective against *E. coli*, *S. aureus*, and *C. albicans* but chloroform and *n*-hexane extracts lacked such inhibition effect. 

Fruits of *Rosa* spp. are generally rich in the bioactive compounds with antioxidant activity, especially in flavonoids, tannins, carotenoids, mineral compounds, phenolic acids, and fatty and organic acids [30]. Results on the total antioxidant activity with DPPH method ranged from 6.99 to 7.73 mg TEAC/g (Table 3). In the study of Gruenwald [31], the antioxidant activity of 0.94 ± 0.03 mmol Trolox equivalents (TE) and the total phenolic contents of 0.080 mg gallic acid equivalents were detected in *R. canina* extract. Taneva et al. [32] reported strong antioxidant activity of *R. canina* fruits of 295 mM TE/g with the DPPH assay. High antioxidant activity (DPPH–87.78%) had been correlated with total polyphenolic contents in study by Fattahi et al. [33]. Tekeli [34] observed the reduction of the oxidative stress induced in broilers by cold after supplementing of the feed with extracts of *R. canina* fruits.

The total polyphenol contents ranged from 2.61 to 6.33 mg GAE/g in the present study. Our results agree with those of Soare et al. [35] and Ozturk Yilmaz and Ercisli [26], who found the total amount of polyphenols in *R. canina* fruits of ~5.16 mg GAE/g and ~1.02 mg GAE/g, respectively. The contents of polyphenols and the antioxidant activity can be influenced by agro-ecological conditions, including locality, humidity, soil composition, water deficit, and temperature. Motsa et al. [36] found that the contents of secondary metabolites, especially of polyphenols, can increase under drought conditions because of the increased oxidative stress perceived. Similarly, Oh et al. [37] revealed that drought stress and high temperatures can lead to increased amount of bioactive compounds which may serve as the main defense mechanism. The content of bioactive compounds can also be affected by genetic polymorphism of the plant. Prior cytological and chemical analyses of medicinal plants, evidenced a reverse relationship between the ploidy level and the phenolic composition of plants, with a comparatively higher amount determined in diploids than in tetraploids [38]. 

The contents of total flavonoids ranged from 0.08 to 2.03 mg QE/g in our study. In the study of Roman et al. [39] the amount of total flavonoids was from 1.01 to 1.63 mg QE/g and in that of Adamczak et al. [40]—0.52 mg QE/g. These findings are comparable with our results. According to Bhave et al. [41] flavonoids, especially glycoside derivatives of quercetin—quercitrin, isoquercitrin also hyperoside and some aglycones such as catechin, taxifolin, and eriodictyol—could be found in rosehip.

The total phenolic acids contents in the tested fruits ranged from 0.18 to 1.15 mg CAE/g. Demir et al. [42] and Elmastaş et al. [43] identified the phenolic acids in rose fruits as gallic acid, 4-hydroxy benzoic acid, caftaric acid, 2,5-dihidroxy benzoic acid, chlorogenic acid, *t*-caffeic acid, *p*-coumaric acid, and ferulic acid. The contents of total carotenoids varied from 37.12 to 99.62 µg/g in *R. canina* fruits in the present study. In the study by Ropciuc [44], the amount of total carotenoids in rose fruits from Suceava district ranged between 90 and 620 µg/g. The main carotenoids in rose fruits were lycopene, ß-cryptoxanthin, ß-carotene, rubixanthin, gazaniaxanthin, and zeaxanthin, according to Koczka et al. [45]. 

Finally, the genome size, ploidity levels and iPBS markers analysis were performed. The determined genome size ranged from 2.3 to 3.08 pg and the ploidity was found to be from pentaploids up to the octaploids (Table 4) and these results were partially reported previously [45]. In the Modra-Pažite locality, hexaploids were the most abundant; in both of the other two localities, the determined ploidy levels were varied. 

Dog roses are well-known for the alloploid architecture of their nuclear genome, a result of autoploidization, apomixis, and hybridization events [46]. Increased gamete ploidy was reported under high temperature [47]. Apomixis at the level of 5% to 10% and hybridization of up to 49% were reported previously in various crosses of dog roses [45,47]. Genome size of dog roses can thus vary and retrotransposition events are thought the main natural factors affecting this. Retrotransposition, activated by abiotic as well as biotic stresses, is well-known for its ability to modify the size of nuclear genome [48]. In the section *Caninae*, variable levels of ploidy were reported widely [49,50,51]. High genomic size variability renders it difficult to be determined precisely [52]. All of the analyzed genotypes from the locality of Vrbové-Baraní dvor were members of the *Canineae* with genome size in the range of 2.3 to 2.69 pg that was in accordance with Roberts et al. [53] who reported genome size values in the range of 2.07 to 3.79 pg and ploidy levels of 4×, 5×, 6×. All of the genotypes analyzed in this study correspond to these values except the one where the determined ploidy level was oktaploid. This value was, however, reported as a possible one by Wissemann [49]. As for the other detected values of ploidy, pentaploidy and hexaploidy were reported previously [54]. Non-hybridogenic dog roses analyzed by Herklot and Ritz [55] were reported to be pentaploids in most cases and hybrids were reported to be hexaploids. Hexaploid synthetic rose hybrids were described previously to be a consequence of unreduced egg cells or pollen grains [56]. Asymmetrical crossing barriers have been already documented for dog roses [57].

**Figure 7:**
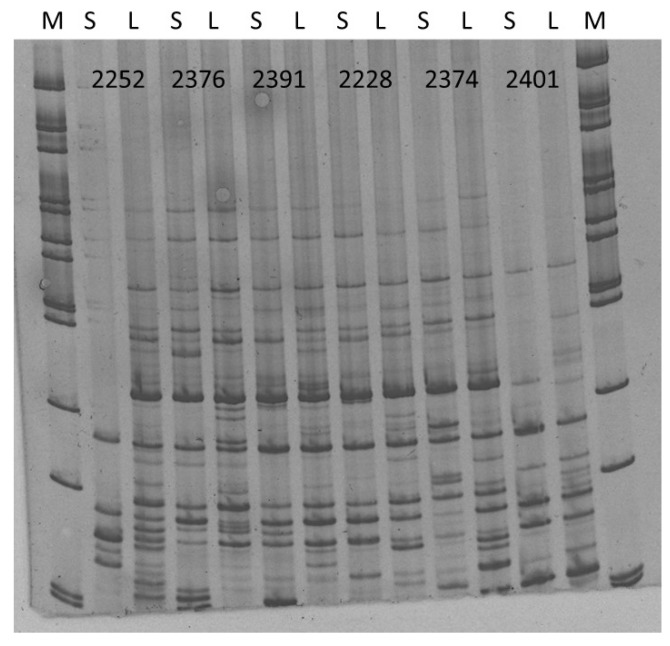
Obtained iPBS amplicon profiles of for *Rosa canina* var. *squarosa* A.Rau (S) and *Rosa canina* var. *lapidicola* Heinr. Braun. (L) when several markers were utilized. M—100 bp ladder. Numbers represent the codes of iPBS markers tested.

Finally, the differences in the iPBS (inter primer binding sites polymorphism) fingerprinting were analyzed for *Rosa canina* var. *squarosa* A.Rau and *Rosa canina* var. *lapidicola* Heinr. Braun collected from Zobor-Lyžiarska lúka, Slovakia. Both of these accessions were deemed pentaploids with comparable genome sizes, hence, the iPBS fingerprint differences should be a consequence of unique retrotransposon insertional pattern of individual dog rose varieties. iPBS amplicon profiles were obtained for a total of six different markers (Figure 7) with specific profiles that allow to distinguish them.

**Figure 8:**
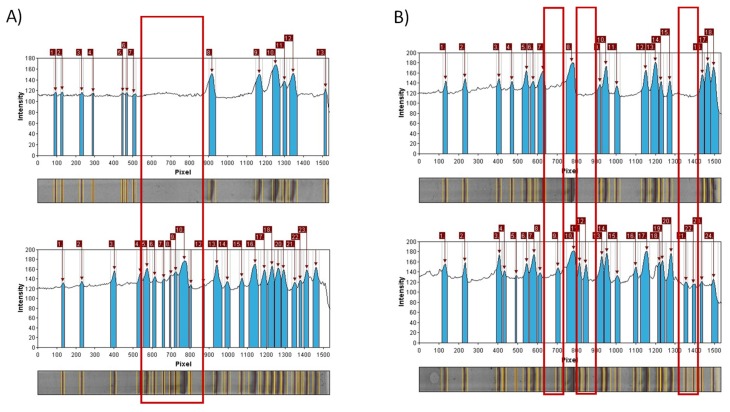
Obtained iPBS unique amplicons regions in *Rosa canina* var. *lapidicola* Heinr. Braun. (L) (down) for markers 2252 (**A**) and 2376 (**B**).

A total of 230 scorable amplicons were generated with many insertions and deletions in loci for both of the analyzed varieties of *Rosa canina*. Clear differences were found in the profiles of those gDNA samples amplified using the iPBS primers. All of the polymorphisms in the iPBS profiles were attributed to specific biological characteristics of retrotransposons. Most of the plant retrotransposons are in the chromosomes as nested, mixed, inverted, or truncated [57]. In all of the analyzed iPBS markers used in this study with the exception of 2374, the fingerprint profile of 120 ng of DNA in PCRs was more abundant for *Rosa canina* var. *lapidicola* Heinr. Braun. with some inserted loci (Figure 8). 

The fingerprinting profiles obtained in the iPBS analyses are in concordance with the basic abundance variability of retrotransposon families and their occurrence in plant genomes [57] and provide a very suitable and effective DNA marker technique for the analysis of plant genomes variability. Up to date, retrotransposon and other DNA markers have been used successfully in the studies of different plant species [57] and here it was proved to be a suitable method for molecular differentiation of *Rosa canina* varieties.

## 3. Materials and Methods 

### 3.1. Plant Materials

The fruits of *R. canina* from non-cultivated plants growing in Slovakia were collected in August 2019 from three places in Modra Pažite GPS 48°20′45″ N 17°19′38″ E (samples, No. 1 to 3), Vrbové-Baraní dvor 48°37′40″ N 17°43′26″ E (sample No. 4), and Zobor-Lyžiarska lúka 48°21′04″ N 18°05′43″ E (sample No. 5), Slovakia. Fruits were picked from bushes, placed in plastic bags, and transported to the laboratory in dark with ice. Plants of the same localities were used for the analysis of genome size and ploidy (see below). 

Taxonomic determination of individual plants of *Rosa* L. was performed directly in the field using the key of European roses, published in Flora of Slovakia [58]. This taxonomic key is based on the determination of the individual botanical characteristics of *Rosa* spp. 

### 3.2. Microbiological Analysis

For the microbiological testing, series of decimal dilutions were prepared from 1 g of *Rosa canina* fruits homogenated with 9 mL of physiological solution (0.89%). For the total viable count (TVC) and mesophilic anaerobic sporulating bacteria (MASB), plate count agar (PCA, Oxoid, Basingstoke, UK) was inoculated with 0.1 mL of suspensions and incubated for 2 days at 37 °C aerobically (TVC) or anaerobically (MASB). For *Pseudomonas* sp., sample suspension was plated out on Pseudomonas Isolation Agar (PIA, Oxoid, Basingstoke, UK). *Pseudomonas* spp. colonies were counted after incubation for 48 h at 30 °C. For Enterobacteriaceae, violet red bile lactose agar (VRBL, Oxoid, Basingstoke, UK) was inoculated with 0.1 mL of suspension and incubated at 37 °C for 24 h. 

For determinations of the microscopic fungi, fruits of *R. canina* were soaked in 9.9 mL of sterile autoclaved water with 0.02% Tween 80 and shook for 30 min. Malt Agar and Czapek-Dox Agar (MA, CDA, Oxoid, Basingstoke, UK) were inoculated with the suspensions and incubated at 25 °C for 5 days. The yeasts were incubated on glucose yeast peptone agar (GYPA, Oxoid, Basingstoke, UK) at 25 °C for 72 h. 

### 3.3. Identification of Microorganisms Using MALDI TOF MS Biotyper

Qualitative determination of endophytic microbiota was performed using MALDI TOF MS Biotyper (Bruker Daltoncs, Bremen, Germany). Sample preparation was carried out according to the MALDI TOF MS Biotyper manufacturer’s recommendations. Briefly, the colonies of bacteria were picked from Petri dishes and transferred into 300 μL of distilled water and 900 µL of ethanol, and the tubes were centrifuged for 2 min at 14,000 rpm. The supernatant was removed, and the centrifugation under the same conditions was repeated for the pellet. All remaining liquid was removed, and the pellet was allowed to dry. After that, 10 µL of 70% formic acid were mixed with the pellet and 10 μL of acetonitrile were added. Tubes were centrifuged for 2 min at 16215× *g* and 1 μL of the supernatant was applied for identification with the MALDI TOF. Matrix, α-Cyano-4-hydroxycinnamic acid in a volume of 1 μL, was added to each 1 µL of supernatant sample and allowed to dry. The analysis was performed using a Microflex LT (Bruker Daltonics, Bremen, Germany) instrument and Flex Control 3.4 software and Biotyper Realtime Classification 3.1 with BC specific software. Confidence scores of ≥2.0 and ≥1.7 were the criteria for successful identification at the levels of species and genus, respectively.

### 3.4. Preparation of Plant Extracts

Sample of 10 g of dried and crushed fruits of *R. canina* was mixed with 100 mL of ethanol (96%, Sigma-Aldrich, Saint-Lois, MO, USA) and allowed to extract for two weeks at room temperature in dark. Then, the solvent was evaporated from the fruit extracts under the reduced pressure at 40 °C (Stuart RE300DB rotary evaporator, Bibby Scientific Ltd, Stone, UK, and vacuum pump KNF N838.1.2KT.45.18, KNF, Germany). For the antimicrobial assay, the extracts were dissolved in 0.1% dimethylsulfoxid (DMSO; Reachem, Bratislava, Slovakia) in concentration of 1024 µg/mL.

### 3.5. Assessment of Antimicrobial Activity with Disc Diffusion Method Against Selected Bacteria Species

There Gram-negative bacteria, *Escherichia coli* CCM 3988, *Klebsiela oxytoca* CCM 2934, and *Pseudomonas aeruginosa* CCM 1960, and three Gram-positive bacteria, *Bacillus cereus* CCM 2010, *Clostridium perfringens* CCM 4435, and *Listeria monocytogenes* CCM 4699, were used for the assessment of antimicrobial activity. The bacteria were obtained from the Czech Collection of Microorganisms. 

For the assessment of antimicrobial activity, test bacteria were grown in 10 mL of Muller Hinton Broth (MHB, Imuna, Slovakia) at 37 °C until they reached a density of approximately 10^5^ cells/mL. Subsequently, 100 µL of the microbial suspensions were spread onto Mueller Hinton Agar (MHA, Oxoid, Basingstoke, UK). Sterilized filter paper discs (Oxoid, Basingstoke, UK) of 9 mm in diameter were impregnated with the 10 µL of dog rose fruit extracts and placed on the inoculated agar plates. The diameters of the inhibition zones were measured in millimeters after 24 h of incubation at 37 °C. All measurements were taken with a precision of 1 mm. Each antimicrobial assay was performed at least in triplicate. Filter discs impregnated with 10 µL of distilled water were used as the negative control, whereas gentamicin (10 µg/disc; Oxoid, Basingstoke, United Kingdom) as the positive control.

### 3.6. Detection of Antibacterial Activity with Minimal Inhibition Concentration (MIC)

*R. canina* fruits were dissolved in DMSO (Sigma-Aldrich, Saint-Lois, MO, USA) to a final concentration of 2048 µg/mL. MICs were determined in Mueller Hinton broth (MBH, Imuna, Slovakia). DMSO *Rosa canina* fruit extracts were prepared as serial two-fold dilutions for concentrations of 1 to 1024 µg/mL. Bacterial suspension of 0.5 McFarland density was used for microplate incubation. Microplates were incubated at 37 °C for 24 h and inhibition of bacterial growth was measured at 570 nm using the Glomax plate spectrophotometer (Promega Inc., Madison, WI, USA).

### 3.7. Sample Preparation for the Assessment of Bioactive Compounds

An amount of 0.2 g of sample was extracted with 20 mL of 80% ethanol for 2 h and centrifugated at 4000× *g* (Rotofix 32 A, Hettich, Kirchlengern, Germany) for 10 min under room temperature. The supernatant was used for analyses of the antioxidant activity and the contents of polyphenols, flavonoids, and phenolic acids. Extraction was carried out in triplicate. 

For the analysis of total carotenoid content, 0.5 g of sample was homogenized in a mortar with sea sand and repeatedly extracted with 10 mL acetone until the sample became colorless. The extract was filtered using Whatman filter paper. Extraction was carried out in triplicate. 

### 3.8. Radical Scavenging Activity—DPPH Method 

Radical scavenging activity of the extracts was measured using 2,2-diphenyl-1-picrylhydrazyl (DPPH, Sigma-Aldrich, Saint-Louis, MO, USA) [59]. The sample (0.4 mL) was mixed with 3.6 mL of DPPH solution (0.025 g DPPH in 100 mL methanol). Absorbance was determined using a 6405 UV/Vis spectrophotometer (Jenway, Stone, England) at 515 nm. Trolox (6-hydroxy-2,5,7,8-tetramethylchroman-2-carboxylic acid) (10–100 mg/L; R^2^ = 0.989; Sigma-Aldrich, Saint-Louis, MO, USA) was used as the standard and the results were expressed as Trolox equivalents in mg/g. 

### 3.9. Total Polyphenol Contents 

The extracts were analyzed using the method with Folin-Ciocalteu reagent (Sigma-Aldrich (Saint-Louis, MO, USA) [60]. A 0.1 mL of sample was mixed with 0.1 mL of the Folin-Ciocalteu reagent, 1 mL of 20% (*w*/*v*) sodium carbonate, and 8.8 mL of distilled water. After 30-min incubation in darkness, the absorbance was measured using a 6405 UV/Vis spectrophotometer (Jenway, Stone, England) at 700 nm. Gallic acid (25–300 mg/L; R^2^ = 0.998) was used as the standard and the results were expressed as gallic acid equivalents in mg/g. 

### 3.10. Total Flavonoid Contents 

Total flavonoids were determined using the modified method of Willett [61]. A 0.5 mL of sample was mixed with 0.1 mL of 10% (*w*/*v*) ethanolic solution of aluminum chloride, 0.1 mL of 1 M potassium acetate, and 4.3 mL of distilled water. After a 30-min incubation in darkness, the absorbance was measured using a 6405 UV/Vis spectrophotometer (Jenway, Stone, England) at 415 nm. Quercetin (0.5–20 mg/L; R^2^ = 0.989) was used as the standard and the results were expressed as quercetin equivalents in mg/g. 

### 3.11. Total Phenolic Acid Contents

Total phenolic acids content was determined according to the Polish Pharmacopoeia [62]. A 0.5 mL of sample extract was mixed with 0.5 mL of 0.5 M hydrochloric acid (Reachem, Bratislava, Slovakia), 0.5 mL of Arnova’s reagent (Reachem, Bratislava, Slovakia) (10% NaNO_2_ + 10% Na_2_MoO_4_), 0.5 mL of 1 M sodium hydroxide, and 0.5 mL of water. Absorbance at 490 nm was measured using a 6405 UV/Vis spectrophotometer (Jenway, Stone, England). Caffeic acid (Sigma-Aldrich, Saint-Louis, MO, USA) (1–200 mg/L, *R*^2^ = 0.999) was used as a standard and the results were expressed as caffeic acid equivalents in mg/g. 

### 3.12. Total Carotenoid Contents 

Petroleum ether (Reachem, Bratislava, Slovakia) was pipetted into a separating funnel with a Teflon cap. The acetone extract of sample and distilled water was added in a manner that allowed them to flow along the walls of the funnel. The mixture was allowed to separate into two phases, and the aqueous phase was discarded. The petroleum ether phase was washed two times with distilled water to remove the residual acetone. The petroleum ether phase was collected in a 50 mL volumetric flask by passing the solution through a small funnel containing 5 g of anhydrous sodium sulfate to remove the residual water. 

The volumetric flask was then filled to its nominal volume with petroleum ether, and the total carotenoids content was determined from the molar absorption coefficient of β-carotene [63]. The concentration (µg/g) of carotenoids was calculated according to the following formula:

(1) $$TCC\;\left[\frac{\mu g}{g}\right]=\frac{A\;\times r\;\times V\;\times 10}{E\;\times n}$$

where: A is the absorbance at 445 nm; r is the sample dilution; V is the volume [mL], E is the molar absorption coefficient E^1%^_1cm_ = 2620; n is the sample weight (after evaporating the petroleum ether); TCC is the total carotenoid content.

### 3.13. Analysis of Genome Size, Ploidy, and iPBS Polymorphism

Genome size and ploidy levels were analyzed using a Partec CAIII flow cytometer (Partec GmbH, Münster, Germany). For the purpose of genome size analysis, the plant material was grown in a greenhouse where the sprouts were induced at 20 °C and 50% humidity for a total of fourteen days. *Pisum sativum* L. variety Ctirad was used as the reference standard with a genome size of 9.09 pg. Method of CyStain PI Absolut P (Partec) was used for the genome size determination by following the manufacturer´s instructions. A total of 0.5 cm^2^ of leaf tissue was used in the analysis. Cytometric data were collected for 5000 nuclei per sample and analyzed in triplicates. Genome size was estimated using the formula given by Doležel et al. [64].

For the purpose of ploidy analysis, the plant material was grown in a greenhouse where young leaves were obtained under the conditions of 22 °C and 55% humidity for a total of fourteen days. *Rosa arvensis* Huds (ploidy of 2n = 14) samples were analyzed simultaneously as the internal standard for each dog rose sample. 

iPBS method was used to determine the differences in the selected samples of *R. canina* collected in the Zobor – Lyžiarská lúka to confirm the changes in the DNA fingerprinting upon detected variability in the ploidy in *Rosa canina* samples. A total of six different iPBS markers were used according the Kalendar et al. [64]. Total genomic was extracted from the at least five young leaves of each sample by GeneJet Plant Genomic DNA Extraction Kit. DNA purity and concentration was assessed using Nanodrop™ (Implen). MyTaq™ Mix with 120 ng of DNA and 600 nM of primers were used under the following PCR thermal profile: 95 °C 4 min; 35 cykles of 95 °C 1 min; 55 °C 1 min; 72 °C 2 min, and the final extension of 72 °C 10 min in BIO-RAD C1000™ Thermal Cycler. Amplified loci were analysed by GelAnalyser softwer.

### 3.14. Statistical Analysis

Data of each replication were averaged, and log transformed. The statistical processing of the TVC, MASB, *Pseudomonas* spp., *Enterobacteriaceae*, microscopic fungi and yeast counts, evaluation of antioxidant activity, and content of total polyphenols, flavonoids, phenolic acids, carotenoids was done with STATGRAPHICS 5 software (Statpoint Technologies, USA). Results were expressed as the mean values with standard deviations (SD) and coefficient of variability (CV). Duncan´s test was applied for the evaluation of antioxidant activity, and the content of total polyphenols, flavonoids, phenolic acids, and carotenoids, as well as the antimicrobial activity of *Rosa canina* extracts. 

## 4. Conclusions

In all collected samples of *Rosa canina* fruits, microbiological contamination was detected with variable levels that depended on the growing locality of the plant. The fruit ethanolic extracts of *R. canina* showed an antimicrobial effect against *Escherichia coli.* Biologically active compounds with antimicrobial and antioxidant activity were found in the *R. canina* fruits, such as polyphenols, flavonoids, phenolic acids, and carotenoids. The *R. canina* endophytic microorganisms seem to play an important role in the production of bioactive compounds. Results of flow cytometry analysis confirmed variability in the genome size and ploidy levels of the members of *Rosa* spp.; these data correspond to the known records from the *Caninae* section. Unique iPBS loci were found that differentiate *Rosa canina* var. *squarosa* A. Rau (S) and *Rosa canina* var. *lapidicola* Heinr. Braun. (L) in their DNA fingerprinting patterns. This study broadens the knowledge of basic dog roses characteristics and a new data about specific antimicrobial characteristics as well as specific iPBS fingerprints are reported.

## Figures and Tables

**Table 1:** Antimicrobial activity of *Rosa canina* fruit extract.

	Extract Activity in Mm				
Microorganisms	1.	2.	3.	4.	5.
EC	14.67 ± 0.58	15.33 ± 0.58	15.67 ± 1.15	15.33 ± 1.15	14.33 ± 0.58
KO	11.33 ± 0.58	11.33 ± 1.15	11.67 ± 0.58	10.67 ± 0.58	12.00 ± 1.00
PA	10.67 ± 0.58	10.33 ± 0.58	10.33 ± 1.15	9.33 ± 0.58	9.67 ± 0.58
BC	8.67 ± 2.31	8.33 ± 0.58	8.67 ± 1.53	8.00 ± 1.00	8.33 ± 0.58
CP	4.67 ± 0.58	5.33 ± 1.53	4.33 ± 0.58	4.67 ± 1.53	4.00 ± 1.00
LM	6.67 ± 1.53	6.33 ± 0.58	5.67 ± 1.15	5.67 ± 0.58	6.00 ± 1.00

EC—*Escherichia coli* CCM 3988, KO—*Klebsiela oxytoca* CCM 2934, PA—*Pseudomonas aeruginosa* CCM 1960, BC—*Bacillus cereus* CCM 2010, CP—*Clostridium perfringens* CCM 4435, LM—*Listeria monocytogenes* CCM 4699. Any of the analyzed sample pairs were statistically different.

**Table 2:** Minimal inhibition concentration of rose fruit extract in µg/mL.

	Extract				
Microorganisms	1.	2.	3.	4.	5.
EC	32	32	32	32	32
KO	64	64	64	64	64
PA	64	64	64	128	128
BC	128	128	128	128	128
CP	512	512	512	512	512
LM	256	256	256	256	256

EC—Escherichia coli CCM 3988, KO—Klebsiela oxytoca CCM 2934, PA—Pseudomonas aeruginosa CCM 1960, BC—Bacillus cereus CCM 2010, CP—Clostridium perfringens CCM 4435, LM—Listeria monocytogenes CCM 4699.

**Table 3:** The results of biological activity of *Rosa canina* samples.

Sample	AA [mg TEAC/g]	TPC [mg GAE/g]	TFC [mg QE/g]	TPAC [mg CAE/g]	TCC [µg/g]
1	6.99 ± 0.14 ^c^	6.33 ± 0.35 ^a^	2.03 ± 0.11 ^a^	1.15 ± 0.09 ^a^	37.12 ± 0.18 ^e^
2	7.63 ± 0.21 ^a,b^	2.95 ± 0.17 ^c,d^	0.35 ± 0.02 ^d^	0.37 ± 0.02 ^b^	57.57 ± 0.22 ^d^
3	7.73 ± 0.11 ^a^	2.61 ± 0.13 ^d^	0.08 ± 0.01 ^e^	0.18 ± 0.01 ^c^	61.07 ± 0.09 ^c^
4	7.41 ± 0.09 ^b^	5.08 ± 0.31 ^b^	0.92 ± 0.05 ^b^	0.26 ± 0.03 ^c^	99.62 ± 0.15 ^a^
5	7.28 ± 0.05 ^b^	3.21 ± 0.12 ^c^	0.71 ± 0.03 ^c^	0.23 ± 0.02 ^c^	75.14 ± 0.31 ^b^

AA—antioxidant activity; TEAC—Trolox equivalent antioxidant activity; TPC—total polyphenol content; GAE—gallic acid equivalent; TFC—total flavonoid content; QE—quercetin equivalent; TPAC—total phenolic acid content; CAE—caffeic acid equivalent; TCC—total carotenoid content; mean ± standard deviation; different letters in column indicate the mean values, which were significantly different.

**Table 4:** Species of *Rosa* L. from analyses localities with the determined genome size and ploidy.

Specie	Locality	Genome Size	Ploidy
*Rosa canina* var. *canina*	Modra-Pažite (accession 1)	2.4–2.72 pg	pentapoid (2n = 35)
	Modra-Pažite (accession 2)	2.43–2.71 pg	hexaploid (2n = 42)
	Vrbové-Baraní dvor	2.57–2.96 pg	hexaploid (2n = 42)
*Rosa canina*	Zobor –Lyžiarska lúka	2.76–2.80 pg	tetraploid (2n = 28)
*Rosa canina* var. *dumalis*	Modra-Pažite	2.42–2.66 pg	hexaploid (2n = 42)
	Vrbové-Baraní dvor (sample 1)	2.3–2.65 pg	oktaploid (2n = 56)
	Vrbové-Baraní dvor (sample 2)	2.52–2.70 pg	pentaploid (2n = 35)
*Rosa canina* var. *squarosa* A.Rau	Zobor –Lyžiarska lúka	2.31–2.51 pg	pentapoid (2n = 35)
	Vrbové-Baraní dvor	2.45–2.67 pg	octaploid (2n = 56)
*Rosa canina* var. *lapidicola* Heinr. Braun	Zobor –Lyžiarska lúka	2.2–2.51 pg	pentapoid (2n = 35)
